# Adaptive Challenges Rising from the Life Context of African-American Caregiving Grandmothers with Diabetes: A Pilot Study

**DOI:** 10.3390/healthcare3030710

**Published:** 2015-08-17

**Authors:** Dana Carthron, Donald E. Bailey, Ruth Anderson

**Affiliations:** 1 School of Nursing, University of North Carolina at Greensboro, Greensboro, NC 27402, USA; 2 School of Nursing, Duke University, Durham, NC 27710, USA; E-Mail: Chip.Bailey@duke.edu; 3 School of Nursing, University of North Carolina at Chapel Hill, Chapel Hill, NC 27599, USA; E-Mail: ruth.anderson@dm.duke.edu

**Keywords:** Grandmothers raising grandchildren, caregiving, African-American, older adults, diabetes, self-management, adaptive leadership, qualitative longitudinal, retention

## Abstract

To understand the challenges arising from the context within which diabetic African-American caregiving grandmothers self-manage their diabetes we used the Adaptive Leadership Framework. Additionally, challenges to retaining this population in a longitudinal study were examined. In this exploratory, longitudinal, qualitative pilot study, data were collected at five time-points over 18 months. We coded the data using content analysis and conducted the within-case and cross-case analyses using data matrices. Lack of awareness of available resources, represented a technical challenge within the life context of these grandmothers and the remaining three themes: family upheaval; priority setting (with subthemes of difficulty meeting basic needs and competing demands); and self-silencing and self-sacrifice represented adaptive challenges. The context of African-American grandmothers’ lives created primarily adaptive challenges that were complex and without immediate solutions. Research is needed to develop culturally and contextually appropriate interventions to help this vulnerable group develop capacity for adaptive work.

## 1. Introduction

As of 2010, an estimated 2.7 million grandparents were raising grandchildren in the United States (USA); 24% were African-American with women overwhelmingly assuming this role [1]. Adding care of grandchildren creates financial burdens for many African-American caregiving grandmothers who already live under severe financial constraints [2]. The biological parent may receive benefits for the child from the state, however, these benefits may not filter to the grandmother. Often the grandmother does not have legal custody of the grandchild, she is therefore not be entitled to welfare benefits, food stamps, *etc*. This means the grandmothers must delve into their own limited benefits or retirement savings to provide for the needs of their grandchildren as well as themselves. In addition to the financial obligations, as the primary caregiver the grandmother is responsible for child rearing, driving them around to activities, attending school functions *etc*.

In addition to the challenges of resuming the role of “parent”, many African-American caregiving grandmothers have chronic illnesses such as diabetes that they must self-manage. Type 2 diabetes is the 7th leading cause of death in the U.S., with a total of $ 245 billion in total costs [3], with African-American women disproportionately burdened from the disease with 25% over the age of 55 [3]. Self-management is defined as engaging activities to promote and maintain personal well-being [4] including activities specific to chronic illness; self-management is integral to preventing complications of diabetes [5]. Caregiving for grandchildren has been found to exacerbate chronic illness such as diabetes [2,6]. Often these grandmothers have multiple caregiving roles while attempting self-manage their diabetes prioritizing the needs of others above their own. Primary caregiving grandmothers often have to other high-demand roles within the family and community in addition to caring for their grandchildren which may have serious implications on the self-management of their diabetes [7]. Multiple studies have found that African-American caregiving grandmothers often have a decreased ability to self-manage diabetes such as inability to purchase diabetes monitoring strips or medications, due to the multicaregiving role [7,8]. Family members and friends often play important roles in diabetes self-management by facilitating or in some cases acting as barriers to self-management processes [9]. Thus, the path to self-management for these women is challenging given the context of their lives. They are caring for their grandchildren at a time when they are most vulnerable to complications of diabetes [10].

The reasons for this caregiving roles include incarceration, substance abuse and mental illness of the biological parents, and may lead to interfamily conflict over parenting styles and finances when the grandmothers use their limited resources to care for the children [11]. Caregiving of a grandchild has also been found to impact the grandmother’s relationship with her significant other who may disagree on how to discipline children or whether they should be providing kinship care at all. Spouses might feel neglected, experience jealousy because the women have less time for them, and complain about a lack of sexual intimacy [12].

Self-management occurs within one’s life context [13] and thus a broader approach to chronic illness management that includes an understanding of the patient’s unique situation is necessary. Yet, there is limited evidence describing how life context impacts African-American caregiving grandmothers with diabetes or other chronic illness. Therefore, the purpose of this study was to understand the context within which these grandmothers are managing their diabetes and the challenges that arise from the context of their lives. The aims of this study were to: (1) describe the context of these grandmothers; (2) identify and describe their challenges; and (3) determine the feasibility of recruiting and retaining African-American primary caregiving grandmothers in a longitudinal study.

### Theoretical Framework

**Figure 1:**
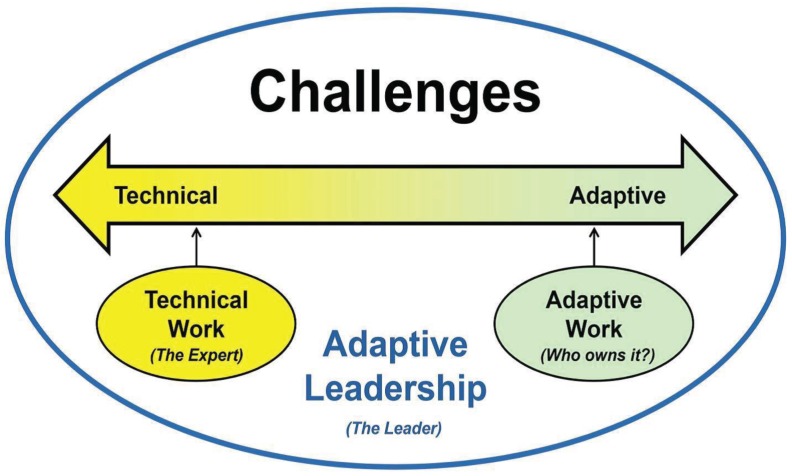
Adaptive Leadership Framework.

The Adaptive Leadership Framework guided the study identifying technical and adaptive challenges [14] as depicted by Anderson *et al*, 2015 [15] (see Figure 1). The framework, when applied to health care differentiates technical challenges that might be addressed by technical work executed by the “experts” (providers or other service workers), from adaptive challenges and the adaptive work that patients must perform for themselves [16]. Patients’ technical challenges, such as an elevated Hemoglobin A1c are considered to be technical, in part, and a “clear-cut solution” exists in established guidelines which the provider follows by ordering medication. Adaptive challenges are those with multiple aspects intertwined making them more difficult to define and often involve work that only the person with the challenge can address. Adaptive challenges require provider or the patient and family to learn new behaviors to address the problem. Examples of adaptive work include diet changes, medication adherence, and stress management. Adaptive work is must be accomplished by the person who owns the challenge. For the diabetic caregiving grandmother this might involve changing lifestyle or practicing stress management. Providers acting as adaptive leaders will do the technical work required but will also engage the patient in addressing his/her adaptive challenges and facility them in gain new skills for self-management. The complex challenges of caregiving, combined with the intricacies of diabetic self-management, demand highly multifaceted and adaptive approaches [17], the hallmark of the Adaptive Leadership Framework [15].

Figure 1 represents our attempt to depict the Adaptive Leadership Framework as proposed by Heifetz *et al*. [14]. Figure 1 suggests that for many problem contexts, challenges likely range on a continuum from purely technical to purely adaptive with most situations having some combination of technical and adaptive challenges. Experts, such as health care providers, address technical challenges using existing solutions but only the person with the adaptive challenge can address the adaptive challenges. Adaptive leadership is the ability to distinguish between technical and adaptive challenges and the ability to align approaches that facilitate the person with the adaptive challenges to gain the skill needed to address his or her challenges.

## 2. Methods

In this exploratory, longitudinal, qualitative pilot study, data were collected at five time-points over 18 months. This method was employed as it is open-ended and intentional linking to the number of waves rather than a single point in time [18]. We developed an interview guide to explore the context within which these grandmothers must try to self-manage their diabetes and the challenges they encountered due to their context. The study was reviewed and approved by the Institutional Review Boards of the universities of the authors.

### 2.1. Sample and Setting

Inclusion criteria were: (1) African-American grandmothers aged 55-years or older; (2) English-speaking, (3) diagnosed with Type-2 diabetes; and, (4) the primary caregiver of at least one grandchild under age 18. Exclusion criteria included caring for someone other than grandchildren in the home.

Interviews were conducted in the grandmothers’ home or a location of their choosing to ensure comfort and feelings of security. The grandmothers decided on the time of day for the interviews, but the PI encouraged them to select a time when the child was either in school or napping to ensure a quiet environment.

### 2.2. Recruitment

Six diabetic African-American primary caregiving grandmothers living in central North Carolina were recruited using purposive sampling. An existing database of grandparents consenting to be contacted for future research studies from the Grandparent Center of Winston-Salem State University was used. The database indicated if the grandmother had diabetes. The Center held regularly scheduled support group meetings; therefore, flyers were posted throughout the Center. We recruited four grandmothers through the Center and two additional grandmothers using snowball sampling, in which grandmothers referred others. Snowball sampling was useful because African-Americans traditionally have been distrustful of researchers [19]. Once identified, potential participants were provided information regarding the study and asked about their interest in participating. After screening for eligibility, written consent was obtained. All participants received a $ 25 Walmart gift card after completing each interview.

### 2.3. Interviews

The investigators developed two interview guides (Table 1), one for the first interview and then one for the four subsequent interviews based on an in-depth review of literature, ADA Standards of Diabetes Care [3] and Carthron’s previous study [6]. The interviews explored the context in which the grandmothers managed their diabetes while caring for their grandchildren using the global question “Tell me what it is like to be diabetic while caring for your grandchildren.” Probes were used to assist the grandmother in elaborating and clarifying statements. The interview guides was refined as needed throughout the study based on analysis of transcripts.

**Table 1:** Interview guide.

1.	Tell me what it’s like to be diabetic while raising your grandchildren?
2.	How has your health changed since you started raising your grandchildren?
3.	Your grandchildren are_______years old. Do their ages impact how manage your diabetes?
4.	How do you know if your diabetes is better or worse?
5.	What doesn’t help or makes it harder to manage your diabetes since raising your grandchildren?
6.	What assists you with the management of your diabetes?
**Subsequent Interviews**	
1.	How has the caregiver role influenced how you check your blood sugar? Checking your feet? Eating a healthy diet? Exercising at least 3 times per week for 30 min? Going to the doctor for your regular checkups? Having your eye checked?
2.	How and why have these changes in self-management activities occurred?
3.	What are your needs regarding checking your blood sugar? (Same self-management activities as above)?
4.	Is there anything else that helps you manage your diabetes better?
5.	Is there anything else that prevents you from managing your diabetes?

### 2.4. Data Collection

All interviews were conducted by the PI approximately every two months for a total of five collection points (Table 2) with two additional interviews occurring during the clinical encounter (to be described in an additional paper). All participants rescheduled at least one interview appointment extending the initial length of the study. The participants became extremely forthcoming toward the end of the study concerning the self-management activities and life context; and, therefore an additional interview was added for all participants as rapport was established. Each was digitally recorded and lasted between 45 min and 90 min. Field notes were used to record contextual information and details about the interview experience; brief notes were jotted during the interview and then expanded and dictated by the interviewer immediately after the interview. The Co-Investigators listened to recordings of the early interviews and together the team adjusted the interview approaches and refined questions to ensure that the data could address the research questions. The research team met weekly to discuss coded data and identified areas for follow up with each participant in subsequent interviews.

### 2.5. Data Preparation

The interviews were transcribed verbatim by a professional service. The PI reviewed each transcript for accuracy by listening to the recording and prepared them for coding in Atlas TI.

**Table 2:** Data collection activities and rationale.

Month	Activity	Rationale
1	Interview to explore the lived experience of being diabetic and caring for grandchild	Previous study revealed that grandmothers wanted to discuss experiences as a caregiver before they were ready to discuss health.
3	Self-management interview & survey of self-management activities	Explore lived experience and identify self-management activities.
5	Provider visit followed by a focused interview with the grandmother about provider support	Observe interaction between provider & grand-mother. What topics were discussed? Was grand parenting role assessed & considered in the visit? Self-management discussed? In the interview, ask questions such as What was helpful, what was not? What were your goals for the visit? Were they met?
7	Same as month 3	Same as month 3 with addition of asking about changes in self-management trajectory.
9	Same as month 5	Same as month 5.
11	Same as month 3	Same as month 7
12	Synthesis interview	To explore any final comments and close relationship with participant

### 2.6. Data Analysis

Data collection, coding, and analysis occurred simultaneously so that all new text was compared with previously coded text [20]. ATLAS TI 6.2 [21], a comprehensive network analysis software package, was used to classify and sort the data. The members of the research team coded the first interview for each grandmother, and identified codes which were refined in weekly meetings. All codes were defined in a code book. Raw data quotes were not altered or changed to ensure the integrity of the findings. Using the code book, the first author coded the remaining interviews and all coding decisions were reviewed by the full research team. To ensure inter-rater reliability, all coding disagreements were resolved with discussion. Each interview was coded and compared to previously coded interviews from earlier data collection points with all data organized in a matrix for each case to facilitate deep understanding of the cases and to facilitate cross case analysis [22]. After reviewing all codes, categories were established and themes extracted.

### 2.7. Scientific Rigor

Multiple techniques were used to address credibility, dependability confirmability and transferability [23]. These are described in Table 3.

**Table 3:** Strategies for assuring rigor of the case study design.

Confirmability—freedom from unrecognized researcher biases Meeting with research team members reviewed data collection and analysis procedures Review of data collection and analysis by team members to assess for study biases Research team member continuously ensured rival hypotheses or conclusions are considered Member checks were used to determine that case reports reflect the realities of the participants
Dependability—the process of the study is consistent across researchers and settings Case study protocols used so that data collector used comparable procedures A code book used to provide for consistency across data analyzers and time points Coding checks assessed level of agreement; disagreements resolved through discussion An audit trail established Investigators assessed connectedness of the study to the guiding conceptual framework.
Credibility—authenticity and plausibility, or truth value of the results Analysis triangulation between multiple forms of data to strengthen inferences Findings compared with current adaptive leadership literature
Transferability—usefulness beyond the individual participants in the study Rich detail of data facilitated comparison of findings in other contexts Explicit criteria for the case selection provided for comparisons with other samples Rich descriptions in the data allowed judgments about potential transferability

## 3. Results

### 3.1. Sample

Each grandmother had at least one grandchild living with her and two were raising grand and great grandchildren simultaneously. The six grandmothers ranged in age from 56 to 74 with the oldest participant raising two grandchildren and a great grandchild. Length of caregiving ranged from 6 to 17 years. Two grandmothers had a 10–12th grade education, two with at least one year of college and two grandmothers were college graduates. Monthly incomes were as low as $ 760 per month and some were greater than $ 2000. All of the grandmothers had some variation of health insurance. Only two grandmothers had legal custody of their grand or great grandchildren. The children’s ages ranged from 12 to 17 and all were in school; however, three did not attend regularly. Although grandmothers initially stated that they were only caring for their grandchildren, all had additional caregiving responsibilities.

#### Adaptive and Technical Challenges

Three main themes described adaptive challenges and one main theme described a technical challenge from the life context of these grandmothers (see Table 4). These challenges are defined and described and supported with a synthesis of the data from the six grandmothers.

### 3.2. Adaptive Challenges

The grandmothers described adaptive challenges, defined as those in which the person must do the work to manage the problem and how the challenges created barriers to self-management of their diabetes. Three themes were evident within the category of adaptive challenges: family upheaval; priority setting (subthemes of difficulty meeting basic needs and competing demands); and self-silencing and self-sacrifice.

**Table 4:** Identified adaptive and technical challenges.

Adaptive Challenges	Examples
**Family upheaval**	Absent biological parent(s) returns
**Priority setting** Difficulty meeting basic needs Competing demands	Rent and utility bills Providing physical or financial care for adult children
**Self-silencing & self-sacrifice**	The need to be strong for their families by refusing to tell family members of health issues and delays in care because of caregiving obligations
**Technical Challenge**	**Example**
**Lack of Awareness**	Navigating the legal system to obtain financial assistance for child support.

#### 3.2.1. Family Upheaval

Family upheaval, defined for this study as changes in normal family processes, unpredictability of family structure and instability of family roles, was a common theme observed across grandmothers. Three grandmothers expressed that the return of absent biological parent(s) produced upheaval in the household. During these transitions the biological parent(s) often interjected in parenting decisions which created chaos and the grandmothers felt they were expected to relinquish their role as “parent.” Although at times it appeared that both the grandmothers and biological parents were working with the child’s best interest in mind, the grandmothers were very angry and resentful toward the biological parent because of the perceived interference with the grandmothers’ parenting role. Two of the grandmothers also voiced resentment toward their grandchildren for listening to the biological parent(s) and/or taking their advice. The negative feelings became agonizingly intense because the grandmothers were also attempting to protect their grandchildren from knowledge about the parent’s behaviors. As one grandmother described:

“And that has been hard for me to watch, because when (Daughter) was needed to raise these children, put input in ‘em and put that nickel or dime on somethin’, she was not there. You see? And he (grandchild) can't see that because I wiped away all the stuff that he would have seen and (would have) been able to use in the equation.”

Family upheaval emerged when the grandmother disapproved of the parent’s behaviors. One grandmother took custody because of the parent’s drug use and promiscuity. She stated that the grandchildren “didn't need to see all that” and that the biological parent “didn't need to bring that in my house.” This situation caused strain between the grandmother and parent. Another grandmother took custody of her grandchild because she felt the parent’s sexual orientation endangered the grandchild.

“You're not the first person in this family to not know what sex you are.” I said, “My problem with it is, if you thought you were sort of different, then you shouldn’t have had these children.”

Four grandmothers had children who were incarcerated and they attempted to keep family members close by having their grandchildren visit the parent in prison, which often added to the grandmothers’ stress. During this study, one grandmother’s grandchild was arrested and placed in jail which further disrupted the family structure.

When asked how they self-managed their diabetes during these “chaotic” times, two grandmothers said that they tended to overeat and one grandmother reported that she often did not have an appetite. All grandmothers reported forgetting their medications more frequently.

#### 3.2.2. Priority Setting

Priority setting, defined as giving one entity more precedence above another, served as a major adaptive challenge to diabetes self-management across grandmothers. Three types of issues called for prioritization; these were basic needs, competing demands, and classified into two subthemes.

##### 3.2.2.1. Difficulties Meeting Basic Needs

Grandmothers struggled to meet basic needs such as rent and utility bills and placed little priority on their own needs. Two grandmothers consistently had challenges paying for utility services and one grandmother lived without heat for over a month. One grandmother often used her own money to supplement her disabled son’s income, further decreasing the funds available to pay household expenses. Another grandmother described having to make the choice between purchasing necessary medications and paying utility bills.

“Well sometime I’m—you know, like I say I hafta stretch what I'm doing so I can get my medicine ’cause I really do need them. Maybe I let one of the bills run or take over to the next month. That’s what I do.”

None of the grandmothers were addressing fully the basic physiological needs for self-care related to their diabetes and they described negative consequences for their health and wellbeing. Simultaneously, they attempted to meet higher level needs such as love and belonging and esteem; not for themselves, but for their grandchildren. These grandmothers described that it was extremely important for their grandchildren to “fit-in” with their peers and used this as an explanation for purchasing the latest clothing styles for their grandchildren despite not meeting basic household needs and their own health needs.

“I got to put more money in (granddaughter)’s clothes than (grandson). He got a whole bunch of clothes now, but some of—all of ‘em he don’t like to wear. They got to match; they got to have this and that to look like his friends. ‘Oh I want the white Nike shoes’, the whatever, the Polo’s. She (granddaughter) says, ‘I want a (designer) purse’—all this.”

School field trips, college tours, prom/graduation and other extracurricular activities incurred costs for these grandmothers. Because of these expenses, several grandmothers had difficulty paying their medical co-payments and thus missed appointments with their care provider. Four grandmothers had copayments ranging from $ 3.00 to $ 6.00; however, they described this as difficult to manage given their limited incomes and the high expense of caring for a grandchild. One grandmother had a copayment of $ 30.00 for her endocrinologist. Although the grandmother was experiencing symptoms of diabetes complications, she delayed care because she was unable to pay. While she understood the importance of self-management of her diabetes, the grandmother stated she had “bigger problems.”

##### 3.2.2.2. Competing Demands

All grandmothers had demands from their families or communities that posed adaptive challenges for their ability to self-manage their diabetes. Not only were the grandmothers providing care for their grandchildren, some also were providing physical care for disabled adult children, financial support for adult children and estranged spouses, emotional support for others. They also were leaders in their community and church. This sense of obligation to care for others created adaptive challenges and impacted their self-management due to fatigue and additional time demands. One grandmother, who cared for her disabled son as well as her granddaughter, stated:

“I make sure that physically, emotionally and all those things are stable for her (granddaughter). She's as well kept as any young lady could possibly be. And everything she needs in her life is there. But my son is my only child and he is coming through his third recovery from being paralyzed (loss of mobility). And, I'm all he's got. That's the way I look at it. And I have to make sure everything's in place for him.”

A majority of the grandmothers stated that they forgot to check their blood sugar because they were too busy or too exhausted*.* Financial obligations to other family members, in addition to caring for grandchildren, limited the money for medical necessities (glucose strips, medications) to engage in self-care activities.

“I've been taking care of her (homeless daughter). Then (my) son in prison I sent him money, so it's everybody at this point, on me.”

Supporting and giving to the community was vitally important to several grandmothers. One grandmother felt it critical that she participate in an upcoming presidential election and recruited other volunteers, assisted with voter registration, prepared mailings *etc*. Although this provided a sense of purpose for her, it also served as a barrier to self-management of her diabetes. She reported that on multiple occasions she forgot to check her blood sugar and ate fast food.

Five grandmothers also described their important roles in their churches, such as ministering to the “sick and shut-in”, Mothers of the Church and Sunday school teachers. These grandmothers felt it was necessary to maintain those roles and participate in church activities despite the other obligations. One grandmother discussed having time consuming activities within the church:

“This month we have a coupla projects goin’ on at church so I'm doin’ stuff for that, too, so I get to bed at one o'clock, one thirty…and back up at six (to go to work).”

While these activities and roles appeared to serve as a source of comfort, they also often created obstacles too because the activities decreased the time available to care for their diabetes.

#### 3.2.3. Self-Silencing and Self-Sacrifice

Self-silencing, defined as repressing feelings and emotions, as well as self-sacrifice, giving of oneself despite the physical, emotional and financial cost, was apparent in all of the grandmothers and posed an adaptive challenge especially with regard to their health. All grandmothers described feeling that they had to be strong for their families regardless of circumstances and competing demands for their time; “falling apart” was not an option. This self-silencing led to the perception that they had no choice but to deal with all adversities without breaking down physically or mentally.

“I don’t fall apart in front of him (son) and I don't fall apart in front of her (granddaughter). And I very seldom show emotions in front of them. I think that's why I don’t focus on my illnesses, because if I focused on ‘em, they would.”

Self-Sacrifice was a recurrent theme among grandmothers. They described refusing to tell family members of health issues and delays in care because of caregiving obligations. On grandmother stated:

“My back's been hurtin’ for four months and I waited until I couldn’t do anything else about it. I just didn't have time or the money to go to the doctor.”

Sacrificing their health and time for others instead of making necessary provider appointments, checking blood glucose and omitting/stretching medications was a theme among all grandmothers.

### 3.3. Technical Challenge

An overarching theme was Lack of Awareness, defined as being uninformed about potentially available resource, was identified as a technical challenge related to the participants’ life contexts. These grandmothers were not knowledgeable about available resources to assist them in resolving some of the challenges they faced. This technical challenge impacted the grandmother as well as other family members. For example, many of the grandmothers were dealing with legal issues. One grandmother did not have legal custody of her grandchild and, therefore had no financial assistance. Another was attempting to help her 16 year old granddaughter (who she is raising) obtain child support for her child (great-grandchild) but had difficulty navigating the legal system. This was considered a technical challenge because these grandmothers would have benefited from information about resources and appropriate support systems.

### 3.4. Feasibility Results: Establishing Trust

Five of the six grandmothers completed the study. One participant died due to an event unrelated to the study. While all of the remaining grandmothers completed the study, retaining the grandmothers was challenging. The PI scheduled interviews times that the grandmothers stated were best for them; however, all of the grandmothers rescheduled their interviews at least four times over the course of the study with one grandmothers rescheduling six times in one month. Most of the grandmothers cited their grandchild’s illness or school disciplinary action (detention or suspension) a primary reasons for rescheduling interviews. Interestingly, most of these missed appointment times with the PI were during the first year of the research relationship. Only one grandmother rescheduled her interview after the one year mark of the study.

Developing trust between the PI and grandmothers took time. It was approximately 10–12 months before the grandmothers appeared to feel comfortable and safe talking with the PI and, subsequently, provided much more detail regarding their life context as well as providing additional details to the “back story.” The interviews increased in duration with the grandmothers often saying “oh, I thought I told you that” and then sharing additional and more detailed information. The increase in trust appeared to occur because of the PI’s continuing presence (she did not give up and go away) and non-judgmental listening.

### 3.5. Limitations

The sample size is a limitation of this pilot study. However, the longitudinal nature of the study as well as the established rapport and reciprocal trust between the researchers and the participants allowing them be more candid with their experiences. We believe this pilot study serves as a foundation for future qualitative studies with larger populations.

## 4. Discussion

The life context of these grandmothers revealed multiple adaptive challenges and a technical challenge. Feelings of resentment toward the biological parent, which brought about family upheaval (adaptive challenge), was also noted in the findings of del Bene’s 2010 [24] study of caregiving grandmothers in which they caregiving grandmothers “consistently expressed intense feelings of anger toward the biological parents”. Attempts by the grandmothers to protect their grandchildren from the biological parent’s inappropriate behaviors often increased the conflict within the family similar to Dunlap, Tourigny and Johnson’s [25] findings. However, the physiological consequences of the stress of family conflict may result in increased blood pressure and heart rate as well as changes in endocrine and immune systems in the caregiving grandmother.

While all grandmothers felt it was important to self-manage their chronic illnesses, these women prioritized other people’s needs above their own. Financial trepidations forced the grandmothers to choose between engaging in self-management activities or keeping their utilities connected, for example, which posed a significant challenge in meeting basic needs of the grandmother and the entire family. Unfortunately, often neither need was met. The competing demands for time due to the caregiving role decreased the amount of time and energy available to devote to self-care activities. This is consistent with Samuel-Hodge *et al*. [8] who found that multiple roles impacted self-management, and subsequently the health, of African American women with diabetes and McEwen’s *et al*. [26] who found a relationship between caregiving demands and higher hgbA1c in women. Many grandmothers stated they forgot to check blood sugars or to take medications because they were too busy or “too exhausted.” These adaptive challenges, combined with older age further, increase vulnerability to complications of their diabetes.

Our theme of self-silencing and self-sacrifice among the grandmothers was consistent with Superwoman/Strong Black Woman Schema [27] which is the perceived obligation to self-silence feelings of distress or vulnerability, to portray an image of strength for families and communities, and to take on the needs of others while neglecting one’s own needs. The significance of building and maintaining relationships with family and community, as was the case of these grandmothers, may have led to self-sacrifice because they might have believed that expressing their opinions and feelings would jeopardize interpersonal connections [28]. This posed an additional adaptive challenge to self-management of diabetes due to fatigue and time constraints. Thus, being a “strong black women” could be an asset as well as a weakness. A potential intervention might be teaching the importance of maintaining balance between taking care of self in order to care for others reinforcing that while being strong might assist in holding the family and community together, it increases vulnerability to comprised health.

The grandmothers’ lack of awareness of resources increased stressed induced stress which is consistent with the literature [29,30]. This technical challenge could be addressed by a systematic linkage to service coordination such as referral to a social worker provider or other health partner. For example, interventions would require a team approach, including a discussion with a clinic’s financial counselor to help the grandmother develop a strategy to continue the follow-up visits to her provider as well as obtain diabetes medications.

We found that developing a trust between the PI and the participants was essential to obtaining complete and frank data about the life context of the participants. The success with retaining participants in this study might be attributed to the PI’s persistence in calling and rescheduling missed appointments. The “comfort level” expressed by these grandmothers later in the study relationship demonstrated bidirectional trust and respect. However, this trust did not develop quickly especially when the grandmother was concerned about information being shared with entities, such as family and social services that might harm or further disrupt their family structure. Qualitative longitudinal studies often “driven by the intellectual projects and ongoing relationships between the researcher and the researched” [18]. Thus, we conclude that maintaining relationships with these women is essential in longitudinal studies and speculate that not establishing a long-term relationship would be a barrier to obtaining information that truly reflects perspectives of this population.

To say that these women were not self-managing their diabetes or were non-compliant is inaccurate. They managed their diabetes within their life context and with the resources available, including time money or the lack thereof. To help this population, we need self-management regimens that fit into their full lifestyle instead of expecting them to fit their lifestyle into prescribed regimen; doing this will require providers to use adaptive leadership, a provider characteristic that has not been traditionally available to these women.

## 5. Conclusions

It is evident that adaptive challenges are overwhelming for these women and that the type of adaptive work they must do has not been addressed in traditional educational interventions for diabetes self-management. Thus, new self-management interventions that address adaptive challenges are needed to facilitate these grandmothers in gaining the skills they need to improve diabetes management and overall health. While these women said that their health was important, the collective health of their family and communities, appeared to be more important. Fully dedicating themselves to their grandchildren, other family members, the community at large and their church, demonstrates that it is important to them to ensure the physical, mental, and spiritual health of others before their own health.

Because many adaptive challenges of self-managing diabetes arise from their life context, these must also be addressed in future intervention work. Interventions are needed that help people develop adaptive skills for incorporating their self-management activities into daily routines to manage their diabetes. It is less apparent, however, how to help these women incorporate their sense of obligation to family and community into healthy adaptive behaviors for self. The women will likely reject anything less and or make changes that are short-lived is these priorities, which they hold foremost, are not addressed. Thus potential interventions might be developed by working directly with this population using participatory research methods to develop acceptable strategies for helping them gain a stronger value of their own health, make better decisions with limited resources, manage competing demands, and cope with ever-changing family dynamics and conflict. We propose that a weakness of available interventions for this population is that they have not guided providers to explore and incorporate the life context of their patients. Interventions must be developed that do not demand that the patient fit their lives into their self-management regimens but rather, guide providers to work with women in this population on setting goals that will fit in their life context instead; this approach requires adaptive leadership. Such interventions much change conventional thinking about goal setting with the diabetic caregiving grandmother. Most current interventions include goals for diabetics to adhere to all self-management activities with the objective of lowering hemoglobin A1c, ultimately improving overall diabetic health. However, new interventions must help this population engage in adaptive work with goals that fit their life context; for example, an initial goal may be to simply acknowledge the importance of self with longer-term goals of chronic illness self-management. Interventions that challenge the strongly held belief that family and community come first, will be rejected. This suggests a need for intervention development using participative research because only members of this population can help researcher to learn ways to address the issues in a way that is acceptable to them. Because of the paucity of evidence regarding the self-management of diabetes among African-American grandmothers these finding may assist in the foundation for future research devoted to sustainable interventions.
